# Chest pain in young people: Is cannabis a risk factor?

**DOI:** 10.4103/0974-2700.66546

**Published:** 2010

**Authors:** Pascal Bilbault, Corina M Duja, Jean Y Bornemann, Claire Kam, Gerald Roul, Jacques Kopferschmitt

**Affiliations:** Division of Emergency and Intensive Care, Faculty of Medicine, Hôpital de Hautepierre, Strasbourg, France; 1 Division of Cardiovascular Diseases, Faculty of Medicine, Nouvel Hôpital Civil, Strasbourg, France

**Figure 1:**
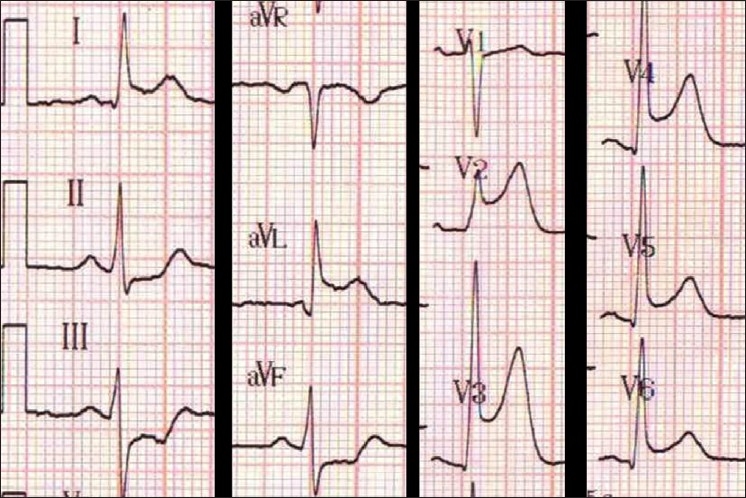
Initial electrocardiogram of Case 1, which shows anterior ST elevation (V2-V4) with reciprocal changes in the inferior leads

Dear Sir,

Cannabis is considered as a common recreational drug generally thought to be a relatively safe substance. However, we have, in the past 6 months, noticed that there is a possible connection between cannabis consumption and coronary disease. We report two cases of young men with no previous medical history admitted to our University Hospital Emergency Department (ED) suffering from acute myocardial infarction (MI) after cannabis smoking 1 h before admission. A further complication occurred in one of them with a cardiac arrest. A 23-year-old man spontaneously presented himself to our ED, suffering from acute chest pain after having inhaled cannabis 1 h before admission. He is a smoker (half a packet of cigarettes daily for 10 years), with a daily consumption of cannabis cigars (at least twice daily). Thorax examination and initial vital parameters were normal. The initial electrocardiogram (ECG) showed sinus rhythm with anterior ST elevation (V2 to V4) with reciprocal ST segment depression in the inferior leads [Figure 1]. Twenty minutes after admission, the patient had a sudden cardiac arrest due to ventricular fibrillation successfully reversed by electrical cardioversion. A coronary angiography was quickly carried out, which showed a unique thrombosis at the initial portion of the anterior interventricular artery. A mechanical desobstruction was successfully performed with stenting, and angiographic control showed no abnormality. The angiogram did not show any obstruction of the other coronary arteries. The initial biological findings were normal, in particular the serum troponine Ic level. Six hours after presentation, the troponine Ic rose from 101 ug/L (normal <0.4 ug/L) to 367 ug/L (after 12 h). The urine drug screen (cocaine, amphetamine, heroine, tetrahydrocannabinol [THC]) was positive only for THC. Clinical evolution was satisfactory, with full recovery, and the patient was discharged 7 days later. Further blood tests showed normal cholesterol and lipid levels. Connective tissue disorder (protein C and S, antithrombin III and cardiolipin) were negative.

**Figure 2:**
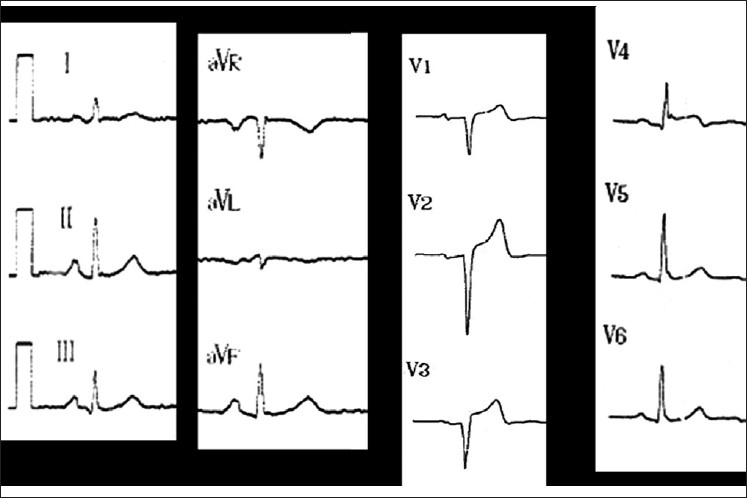
Electrocardiogram of Case 2, which shows anterior ST elevation (V2-V3)

A 29-year-old man spontaneously presented himself to our ED 1.5 h after cannabis smoking with chest pain radiating to his left shoulder. He is a smoker (20 cigarettes a day for 10 years), using cannabis daily. He has a positive family history of hypercholesterolemia. The initial ECG showed sinus rhythm with anterior ST elevation (V2 to V4) [Figure 2]. The initial serum troponine Ic level was at 1.1 ug/L (normal <0.4ug/L) and increased to 320 ug/L 6 h later. The urine drug screen was positive only for THC. Coronary angiogram showed a single thrombosis at the initial portion of the anterior interventricular artery. A mechanical desobstruction was successfully performed with stenting. The patient was discharged 5 days later. As for the previous patient, further blood tests investigating coronary risk factors were negative.

Marijuana is the most widely used illicit drug in the Western countries. It has several well-known side-effects on the cardiovascular system, documented for three decades, and cannabis is suspected to cause sudden death.[1] In a recent report, Caldicott *et al*.[2] reviewed 43 patients affected by acute cardiovascular events associated with cannabis consumption and, among them, only six patients had MI. In the Determinant of Myocardial Infarction Onset Study (3882 patients), the risk of MI onset was multiplied by 4.8-times above the baseline in the first hour after the use of marijuana.[3] However, the risk is less than the one observed with cocaine use (24 times), most likely due to more sympathetic stimulation.[4] More recently, in a prospective cohort study of events after MI with an average follow-up of 3.8 years, Mukamal *et al*. found a mortality risk 4-times greater in cannabis users than in non-users in an age- and sex-adjusted model.[5] Several mechanisms possibly explain why the drug can induce MI risk differently to atherosclerosis. In particular, smoking marijuana in a moderate dose is associated with a stimulation of sympathetic activity leading to an increase in the pulse rate and blood pressure with a decrease in the left ventricular ejection time. At high doses, the parasympathetic activity will also increase, leading to bradycardia and hypotension.[3] Moreover, marijuana induces an increase of carboxyhemoglobin, leading to decreased oxygen-carrying capacity. This carboxyhemoglobin level is higher in cannabis users than in cigarette smokers.[3] This high level induces an increase in the myocardial oxygen demand, with a simultaneous decrease in the oxygen supply. These adverse changes can trigger plaque rupture in vulnerable patients or may induce coronary vasospasm in the presence of normal coronary arteries.[1]

In conclusion, several authors clearly indicate that cannabis smoking could be a trigger for cardiac ischemic events in young healthy users.[6] Even though this risk factor is not as high as that in cocaine users, emergency physicians should not underestimate cannabis-addicted patients complaining of acute chest pain.
